# Obesity and Infection: What Have We Learned From the COVID-19 Pandemic

**DOI:** 10.3389/fnut.2022.931313

**Published:** 2022-07-22

**Authors:** Emilia Vassilopoulou, Roxana Silvia Bumbacea, Aikaterini Konstantina Pappa, Athanasios N. Papadopoulos, Dragos Bumbacea

**Affiliations:** ^1^Department of Nutritional Sciences and Dietetics, International Hellenic University, Thessaloniki, Greece; ^2^Allergy Department, Carol Davila University of Medicine and Pharmacy, Bucharest, Romania; ^3^Allergy Department, Nephrology Hospital Dr Carol Davila, Bucharest, Romania; ^4^Department of Cardio-Thoracic Medicine, Carol Davila University of Medicine and Pharmacy, Bucharest, Romania; ^5^Department of Pneumology and Acute Respiratory Care, Elias Emergency University Hospital, Bucharest, Romania

**Keywords:** adipose tissue, COVID-19, obesity, mortality, infection, severity

## Abstract

**Objective:**

The critical role played by the nutritional status in the complications, duration of hospitalization and mortality in severe acute respiratory syndrome coronavirus 2 (SARS-CoV-2) infection (COVID-19) has emerged from several research studies in diverse populations. Obesity has been associated with an increased risk of serious complications, as the adipose tissue appears to have significant effects on the immune response. The aim of this narrative review was to investigate the relationship between COVID-19 and obesity.

**Methods:**

We performed a review of papers in the English language derived from PubMed, Science Direct, and Web of Science. The primary outcomes investigated were the severity of the disease, admission to the intensive care unit (ICU), need for intubation, and mortality.

**Results and Conclusion:**

Review of 44 eligible studies from 18 countries around the world revealed evidence that obesity increases the risk of severe COVID-19 complications, ICU admission, intubation and mortality. Patients with a higher body mass index (BMI) appear to be more vulnerable to SARS-CoV-2 infection, with more severe illness requiring admission to ICU and intubation, and to have higher mortality. A healthy body weight should be targeted as a long-term prevention measure against acute complications of infection, and in the event of COVID-19, overweight and obese patients should be monitored closely.

## Introduction

On 31 December 2019 the World Health Organization (WHO) was informed by Chinese officials of dozens of cases of pneumonia of unknown etiology in Wuhan, China.

On 11 March 2020 the WHO announced that the whole world was living under the threat of the severe acute respiratory syndrome coronavirus 2 (SARS-CoV-2) epidemic (COVID-19). The response from the scientific community was to produce anti-SARS-CoV-2 vaccines, and rapid immunization of the population was instituted, aimed at halting the spread of the virus.

By April 2022 the WHO reported approximately 504 million confirmed cases of COVD-19, and 6.2 million deaths (1). At this stage, ~59% of the world population had received one dose of COVID-19 vaccine (2). The SARS-CoV-2 has mutated over time, resulting in genetic variations in the population of circulating viral strains over the course of the COVID-19 pandemic (3), and changing health effects, in terms of contagiousness, severity and symptoms (4).

The critical role of the nutritional status on the complications, length of hospitalization and mortality from SARS-CoV-2 viral infection has emerged from several research studies conducted from different perspectives. Weight status is indicated as a significant factor, with obesity being associated with an increased risk of serious complications (5), as the adipose tissue appears to have significant effects on the immune response (6).

Efforts made by the research community to interpret the mechanisms that lead to an exaggerated inflammatory response focus primarily on the role of adipose tissue in the function of white blood cells (WBCs). Specifically, the main mechanisms reported are the following:

### Expression of Angiotensin 2 Enzyme in Adipose Tissue

SARS-CoV-2 has been reported to penetrate human cells by direct binding to ACE2 receptors on the cell surface. The concentration of ACE2 is higher in adipose tissue than in lung, thus suggesting that adipose tissue may be more vulnerable to COVID-19 infection. The obese population has more adipose tissue and higher levels of ACE2, which implies an increased susceptibility to infection due to exaggeration of the inflammatory response (7).

### Metabolic Dysfunction

Human adipose tissue is known for its basic functions in helping to maintain the body's homeostasis, which include energy storage in the form of fat, and supply of energy in prolonged conditions of reduced food intake (8), thermal insulation of the body (9), absorption of environmental vibrations, and mechanical facilitation of the movement of the skin on the underlying tissues (10). In addition, adipose tissue is known to be actively involved in inflammation and immunity (11). Specifically, white fat cells produce numerous endocrine, paracrine and neuroendocrine signals through the production of adipokines. As described above, SARS-CoV-2 penetrates human cells by direct binding to ACE2 receptors on the cell surface (12). Because of the high concentration of ACE2 in adipose tissue, obese individuals may be more vulnerable to COVID-19 infection (13).

Tumor necrosis factor-α (TNF-α) is a multifunctional cytokine involved in many different pathways in human homeostasis and pathophysiology. In animal models, TNF-α is expressed in adipose tissue and affects insulin-induced signaling, inhibiting expression of glucose transporter type 4 (GLUT-4), resulting in raised levels of free fatty acids (FFA), and finally increasing insulin resistance (IR) (14). This promotes the activation of NF-kB, a transcriptional activator that controls proinflammatory cytokine synthesis and cell survival, inducing the pathway to cell death (15).

Excess FFAs, in turn, activate immune pathways of inflammation, through various signaling pathways that promote increase of TNF-α, interleukin-6 (IL-6), leptin, and resistin (16), which act directly on the differentiation of monocytes into activated M1 macrophages. M1 macrophages produce inflammatory cytokines, active oxygen radicals and nitric oxide (NO), affecting the endogenous immune response to pathogens (16). Obesity therefore leads to an inflammatory response characterized by increased cell aggregation and higher production of cytokines.

### Thymus Gland

The thymus gland is an organ characterized by T-cell growth, and any thymus defect or impairment of the production of thymocytes, can lead to profound primary T-cell immunodeficiency (17). Defects affecting both T-cell and B-cell lines result in severe combined immunodeficiency syndrome (SCIDs) (17). Obesity causes a decrease in T-cell receptors (18).

In addition, the peripheral immune response to obesity reduces the migration of antigen-presenting cells (APCs) to peripheral lymph nodes, and lowers the number of T cells, resulting in a reduced immune response against infectious agents (19).

### Adipocyte Function

Obesity, due to the direct evolutionary relationship of metabolic and immune pathways, causes major changes in the number, phenotype and tissue distribution of adipose tissue adipocytes, and in the attraction of various different immune cell populations (20, 21). Thus, about half of the adipose tissue cells in obese individuals are macrophages, characterized by the inflammatory phenotype of classically activated macrophages (M1), while in lean individuals, the macrophage phenotype of adipose tissue is that of anti-inflammatory alternatively activated macrophages (M2) (22). M1-macrophages produce a number of inflammatory agents, including TNF-α, IL-6, IL-1β, chemokine ligand-2 (CCL2), and macrophage inhibitory factor (MIF), resulting in the development of local inflammation (23). Strong experimental evidence suggests that other myelogenous immune cells (dendritic and squamous cells, granulocytes, and granulocytes) contribute significantly to the severe disruption of the network of immune mechanisms in the adipose tissue of obese subjects (24).

The aim of this narrative review was to investigate the association of obesity with the severity and complications of COVID-19 infection, admission to the intensive care unit (ICU), need for intubation, and mortality.

## Materials and Methods

A literature review was carried out, based on a search in PubMed, Science Direct and Web of Science to identify peer-reviewed studies, published between January 2020 and April 2022, using the following keyword combinations: Obesity OR visceral fat OR adipose tissue OR Body Mass Index AND COVID-19 (SARS-CoV-2) severity OR COVID-19 (SARS-CoV-2) complications OR COVID-19 (SARS-CoV-2) intubation OR COVID-19 (SARS-CoV-2) mortality. Additional articles were identified through reference lists of the retrieved articles.

The search was completed on January 10th 2022 and updated on April 15^th^ 2022.

Inclusion criteria were clinical studies that: (1) were quantitative (25) empirical human studies (26); (2) reported obesity prevalence and outcomes in patients with COVID-19; (3) were published in English in peer-reviewed journals. The exclusion criteria were (1) intervention studies with food supplements; (2) reports of patients with other comorbidities (e.g., cancer, cardiovascular disease); (3) studies focusing on other co-factors associated with complications, such as smoking, alcohol; (4) qualitative studies; (5) limited access; (6) reviews, systematic reviews, opinion articles, editorials.

Titles and abstracts were reviewed independently by two researchers (EV, RSB), and discrepancies were resolved through discussion or involvement of the other three researchers (AKP, AP, DB). The data extracted for review included first author's name, country where the study was performed, study type, patient characteristics, weight status and clinical outcomes, including disease severity and complications, admission to the ICU, intubation and mortality. The literature review was conducted with the RAYYAN online tool for systematic reviews (27).

**Figure 1:**
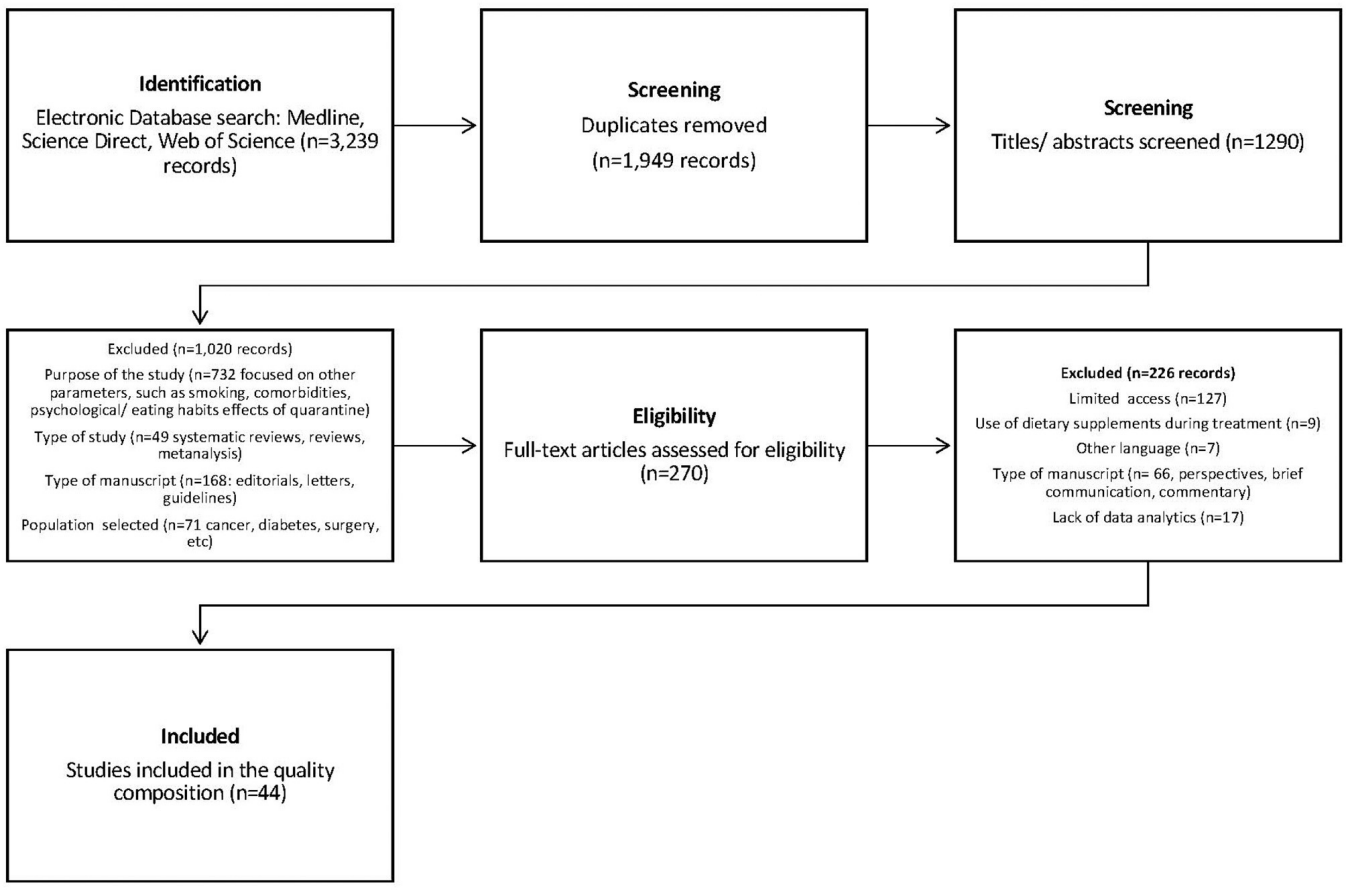
Flow chart showing selection procedure for studies on obesity and COVID-19 infection.

Figure 1 shows the flow chart of the selection of studies included in this review. After removal of duplicates, 1,949 papers were evaluated, of which 44 met the inclusion criteria, and were the subject of the review.

### Quality Assessment

The 44 studies were read extensively and scored according to the Ottawa Scale for case series, cohort, cross-sectional and case-control studies, for each of the following quality markers: (1) the target population was defined clearly; (2) recruitment was complete, random or consecutive; (3) the confidence intervals (CI) or standard error (SE) were reported.

The primary aim of the review was investigation of the association of obesity in patients with COVID-19 with the disease outcome, specifically severity, complications, ICU admission, intubation and mortality.

## Results

The characteristics of the studies included in the review are presented in Supplementary Table 1 and are discussed in detail below.

### Obesity in Patients With COVID-19 and Admission to the ICU

Alkhatib et al. reported on the body mass index (BMI) in a sample of 158 African-American patients with COVID-19 admitted sequentially to a tertiary care center between March 12 and April 9 2020. The BMI was significantly higher in those patients who needed to be hospitalized in the ICU (36.5 vs. 31.9 kg/m^2^, *p* = 0.002). The multiple regression (MR) model indicated that, after testing for all other variables in the model, BMI functions as an independent prognostic factor for ICU admission, with adjusted odds ratio (aOR): 1.115; 95% CI: 1.052–1.182, in African Americans. The predicted aOR indicated that for an increase in BMI by 5 and 10 kg m^2^ (aOR: 1.72; 95% CI: 1.29–2.31 and aOR: 2.97; 95% CI 66–5.32, respectively), the relative probability of admission to the ICU increases by 1.7 and 3 times, respectively (28). Similarly, Palaiodimos et al. in a retrospective study of the first 200 patients with confirmed COVID-19 infection admitted *via* the emergency department (ED), showed that a BMI of 35 kg/m^2^ or higher was independently related to in-hospital mortality (aOR: 3.78; 95% CI: 1.45–9.83; *p* = 0.006), increased oxygen demand (aOR: 3.09; 95% CI: 1.43–6.; *p* = 0.004) and intubation (aOR: 3.87; 95% CI: 1.47–10.18; *p* = 0.006) (29).

In the study of Kalligeros et al. which included all adult patients (≥18 years) with confirmed SARS-CoV-2 virus infection admitted to Rhodes Island General Hospital or Mirian Hospital in Rhodes Island, Greece from February to April 2020, class 2 obesity (BMI ≥ 35 kg/m^2^) was associated with an increased risk of admission to the ICU (aOR: 5.39, 95% CI: 1.13–25.64) (30). Shuelter-Trevisol et al. reported that obesity was an independent prognostic factor (aOR: 6.83; 95% CI: 1.93–24.25) for admission to the ICU, in a sample of 211 patients who were diagnosed with COVID-19 infection between March and July 2020, and were admitted to hospital in the Tubarão area, Santa Catarina, Brazil (31). In the retrospective study of Busetto et al. of 92 patients admitted to a COVID-19 ward from March to April 2020 in Veneto, Italy, admission to ICU or semi-ICU was needed for 18.7% of patients in the normal weight group, 54.8% of overweight patients and 41.3% of patients with obesity (*p* < 0.05) (32).

Similar findings were reported by Argenzian et al. from retrospective study of the first 1,000 consecutive patients with SARS-CoV-2 acute respiratory distress syndrome (ARDS) presenting at the ED or admitted to hospital between March and April 2020 in New York. Specifically, patients admitted to the ICU had a higher rate of obesity than those not requiring intensive care (45.7 and 39.5%, respectively) (33).

In a retrospective cohort study with 14,625 patients in Turkey, Sahin et al. observed that hospitalization, ICU admission, intubation/ventilation, lung involvement and mortality were significantly higher in overweight and obese patients. On adjusted analysis, overweight (OR, 95% CI: 1.82, 1.04–3.21; *p* = 0.037) and obesity (OR, 95% CI: 2.69, 1.02–1.05; *p* < 0.001) were associated with a higher rate of intubation/mechanical ventilation, but only obesity was associated with increased mortality (OR, 95% CI: 2.56, 1.40–4.67; *p* = 0.002) (34).

In Germany, Dreher et al. compared the clinical features of the first 50 COVID-19 patients hospitalized with or without ARDS. Patients with ARDS were more commonly overweight or obese [83% (64, 93) vs. 42% (26, 61)] or had a history of respiratory diseases [58% (95% confidence interval: 39, 76) vs. 42% (26, 61)] (35).

A study from England with 6,910,695 patients conducted by Gao et al. showed a significant positive linear association between each unit of increase in the BMI and admission to the ICU due to COVID-19 (36).

Le Guen et al. retrospectively analyzed data on 600 patients, showing that obese patients had an increased rate of ICU admission (*p* = 0.0215) and increased duration of hospitalization (*p* = 0.0004) (37).

Al Sabah et al. reported that, in a retrospective cohort study of 1,158 patients, the median BMI of patients admitted to ICU was significantly higher than that of those who did not require ICU admission [median BMI: 27.5 kg/m^2^, interquartile range (IQR) 25.3–31.4 kg/m^2^ vs. 26 (23–29) kg/m^2^, respectively, *p* < 0.001] (38).

In another retrospective study, Boudou et al. included 47,265 laboratory-confirmed cases of symptomatic COVID-19 infection from February to November 2020. They concluded that severe obesity, indicated by BMI ≥ 40, was a significant marker for ICU admission (OR 19.6), and a particularly significant predictor among patients with COVID-19 aged < 41 years for ICU admission and < 63 years for death (39).

Cordova et al. in a cohort study of 809 patients treated in 19 hospitals in Argentina, concluded that ICU admission was significantly correlated with male gender (OR 1.81; 95% CI 1.16–2.81), hypertension (OR 3.21; 95% CI 2.08–4.95), obesity (OR 2.38; 95% CI 1.51–3.7), and oxygen saturation ≤ 93% (OR 6.45; 95% CI 4.20–9.92) (40).

Yoshida et al. also in a retrospective study, conducted from February to July 2020 with 776 patients with COVID-19, demonstrated on multivariate analyses, an association between obesity and increased odds of intermittent mandatory ventilation (IMV) and ICU admission (41).

In the study of Wang et al. involving 297 patients with COVID-19 treated sequentially in 10 hospitals in China's Jiangsu Province from January to February 2020, the relationship between BMI level and probability of admission to ICU was not confirmed, as the percentage of patients admitted to ICU was similar in the three BMI groups (normal weight, overweight, obese) (*p* = 0.087). The mean number of days of hospitalization, however, differed between the 3 groups of patients (*p* = 0.025) (42).

### Obesity in Patients With COVID-19 and Disease Severity/Complications

A retrospective study of Rao et al. of 240 patients with COVID-19 admitted to the Union Hospital in Wuhan, China between December 2019 and March 2020, led to the conclusion that patients with severe disease had significantly higher BMI and were more likely to be overweight [*n* = 73 (60.8%) vs. *n* = 41 (34.2%), *p* < 0.001]. In addition, overweight patients were more likely to develop severe pneumonia than normal weight patients [*n* = 73 (64.0%) vs. *n* = 47 (37.3%), *p* < 0.001], and MR analysis indicated that being overweight [aOR: 3.075 (1.187–7.965), *p* = 0.021] is an independent prognostic factor for the development of severe pneumonia (43).

The retrospective study of Deng et al. in 96 patients hospitalized with SARS-CoV-2 infection at Dongguan People's Hospital, Nanfang Hospital and Xiamen University Collaborating Hospital in China in January and February 2020, reported that after treatment, the symptoms had improved or stabilized in 66/96 patients (44). The proportion of patients with a stable disease course with BMI < 24, 24–27.9 and ≥ 28 kg/m^2^ were 74.2 19.7, and 6.1%, respectively, and these rates were significantly different from those of patients with other infections (63.3, 46.7, and 20.0%, respectively, *p* = 0.001) (44).

Negative findings for viral pneumonia on computerized tomography (CT) scan were reported in 85.7, 14.3, and 0%, respectively, of patients with BMI < 24, 24–27.9, and ≥ 28 kg/m^2^, while pneumonia was diagnosed in 54.7, 32, and 13.3% of patients in the respective BMI groups (*p* = 0.027) (45). Obesity emerged as an important prognostic factor at admission for the development of severe COVID-19 disease. In the study of Denova-Gutiérrez et al. obesity was associated with a 1.43 times higher likelihood of developing severe disease (46). Wang et al. (42) showed that the disease severity was dependent on the weight status: severe disease developed in a higher proportion of overweight patients than in those who were normal weight (2.86%, *p* = 0.006), and in obese patients compared with those of normal weight (25 vs. 2.86%, *p* < 0.001). MR analysis indicated that overweight (aOR, 4,222; 95% DE: 1.322–13.476, *p* = 0.015) and obese patients (aOR, 9,216, 95% DE: 2,581–32,903, *p* = 0.001) had an increased chance of developing severe disease after controlling for the effect of the other variables on the model (42).

Hu et al. in a retrospective study of 323 patients with COVID-19 who were hospitalized from January to February 2020 at a reference hospital in Wuhan, observed that patients with BMI ≥ 30 kg/m^2^ were more likely to have an adverse than a positive disease outcome (10.7% vs. 2.9%, *p* = 0.029) (47).

Likewise, Cai et al. (48) in a retrospective cohort study of 383 consecutive patients with COVID-19 admitted from January to February 2020 and monitored until March 2020 at Shenzhen 3rd People's Hospital (China) reported that overweight or obese patients were more likely to have severe disease; 19.2% in the normal weight group, 29.3% in the overweight group, and 39% in the obese group (*p* ≤ 0.001). Overweight patients were 1.84 times more likely to develop severe COVID-19 (aOR: 1.84, 95% DE 0.99–3.43, *p* < 0.05), and those who were obese were 3.4 times more likely to develop severe disease than normal weight patients (aOR: 3.40, 95% DE: 1.40–2.86, *p* = 0.007) (45, 48).

Deng et al. in 65 patients with COVID-19 infection, aged 18–40 years and admitted consecutively from March to April 2020 to the Zhongnan Hospital of Wuhan University in China, observed that patients with severe disease were either overweight (33.3%) or obese (66.67%), and MR analysis showed that high BMI, and especially obesity, is a factor exacerbating the disease severity of COVID-19 infection in young people (44). Similarly, Ioannou et al. in a cohort study involving 101,301 U.S. veterans with SARS-CoV-2 infection, observed that being overweight (BMI 25–29.9 vs. 18.5–24.9 kg/m^2^: aLE, 0.90; 95% DE, 0.77–1.06) or type I obese (BMI 30–34.99 vs. 18.5–24.9 kg/m^2^: aLE, 0.84; 95% DE, 0.69–1.01) or type II obese (BMI> 35 vs. 18.5–24.9 kg/m^2^: aLE, 0.97; 95% DE, 0.77–1.21) was associated with an increased risk of adverse outcome (49).

Nachega et al. in a retrospective study of 766 patients treated for COVID-19 between 10 March 2020 and 31 July 2020 at seven hospitals in Kinshasa, Democratic Republic of the Congo, observed that obese patients were less likely to improve compared with non-obese patients (ASL = 0.27, 95% DE: 0.12–0.59) (50). Fresán et al. conducted a prospective cohort study in the period March to April 2020, in the Navarre region of Spain with 433,995 participants aged 25 to 79 years who had public health insurance. They concluded that those with severe obesity (BMI ≥ 40 kg/m^2^) had a 2.3 times higher risk of developing severe COVID-19 disease [aQR: 2.30 95% CI: 1.20–4.40, *p* = 0.012), after adjusting for other confounding factors in the model (51).

In addition, Hendren et al. analyzed data from patients hospitalized with COVID-19 in 88 US hospitals enrolled in the American Heart Association (AHA) COVID-19 Cardiovascular Disease Registry. Data collected during July 2020 indicated that severe obesity (BMI ≥ 40 kg/m^2^) was associated with an increased risk of in-hospital mortality only in those aged ≤ 50 years [hazard ratio, 1.36 (1.01–1.84)]. In the adjusted analysis, higher BMI was associated with venous thromboembolism and with dialysis initiation, but not with major cardiovascular events (52).

Pantea Stoian et al. investigating the link between mortality and comorbidities, gender, age and hospital pneumonia, showed that obesity is a negative marker for the severity of COVID-19 infection in adults aged ≤ 50 years (*p* = 0.0001) (53).

Terada et al. conducted a cohort study of 3,376 patients, categorizing them into two groups based on the severity of the infection at the time of admission: 2,199 cases (65.1%) were non-severe, and 1,181 cases (34.9%) were severe, and observed that obesity had a major effect on the severity of symptoms (OR 1.75; 95% CI 1.26–2.45, *p* = 0.001) (54).

Finally, Yanover in a cohort of 4,353 patients, studied the complications of COVID-19 in the presence of other comorbidities. Obesity was a risk factor for patients 18–50 years old (OR 11.09, 95% CI 4.15–32.67; *p* ≤ 0.001), while for older patients (50–65 years) a risk factor was chronic kidney disease (OR 4.06, 95% CI 1.89–8.38; *p* = 0.005), while for patients ≥65 years was the neurological disorders (OR 2.65, 95% CI 1.69–4.17; *p* = 0.001) (55).

### Obesity in Patients With COVID-19 and Likelihood of Intubation

Palaiodimos et al. reported that patients with severe obesity were more likely to undergo intubation (BMI < 25 kg/m^2^: 18.4%, BMI 25–34 kg/m^2^: 16.4%, BMI ≥ 35 kg/m^2^: 34.8%, *p* = 0.032). Overall, 45% of the patients had increasing oxygen requirements during their hospital stay, but with no significant differences between BMI categories (56). Simmonet et al. in a monocentric retrospective study involving 124 patients who were admitted to the ICU with confirmed SARS-CoV-2 infection at Roger Salengro Hospital at the “Center Hospitalier Universitaire de Lille” (CHU 27), concluded that the distribution of BMI categories differed significantly between those who needed mechanical ventilation and those who did not (*p* < 0.01, Fisher for trend accurate test). Obesity (BMI > 30 kg/m^2^) and severe obesity (BMI≥ 35 kg/m^2^) were more common in patients who needed mechanical ventilation than in those who did not (56.4 vs. 28.2% and 35.3 vs. 12.8%, respectively). MR analysis showed that being obese (BMI ≥ 35 kg/m^2^ was an independent prognostic factor (aOR: 1.69, 95% DE: 0.52–5.48, compared to reference category BMI < 25 kg/m^2^) for the need for mechanical ventilation, after controlling for the other variables in the model (57).

The retrospective cohort study of Nakeshbandi et al. involved 504 patients screened for COVID-19 at SUNY Downstate Health Sciences University in New York, which was designated by Governor Andrew Cuomo as the COVID Reference Hospital for 10 March 2020 and 13 April 2020. They concluded that overweight patients (RR 2.0, 95% CI 1.2–3.3, *p* = 0.01) and obese patients (RR 2.4, 95% CI 1.5–4.0, *p* = 0.001) had an increased risk of intubation compared with the control category of normal weight (58). Cai et al. reported that the rates of BMI < 24, 24–27.9 and ≥ 28 kg/m^2^ were 52, 24, and 24%, respectively in patients with ARDS, and differed significantly from the rates in those who did not develop ARDS (64.8, 29.6, and 5.6%, respectively, *p* = 0.035) (45). Busetto et al. reported that assisted ventilation (non-invasive and IMV ventilation), in addition to pure oxygen support, was used in 15.6% of normal weight patients, 54.8% of overweight patients, and 41.4% of obese patients with COVID-19, with the difference between the groups being statistically significant (*p* < 0.01) (32).

Klang et al. in a retrospective study of 3,406 patients with COVID-19, admitted to the University Hospital in New York during the period March to May 2020, concluded that the need for intubation and IMV was independently associated with BMI ≥ 40 kg/m^2^, in both the younger (<50 years; aOR 4.1; 95% CI: 2.1–8.2) and the older age group (≥40 years; aOR 1.5; 95% CI: 1.1–2) (59).

In a retrospective study conducted by Deng et al. in 65 patients aged 18 to 40 years, consecutively admitted with COVID-19 infection to the Zhongnan Hospital of Wuhan University in China during March and April 2020, patients with severe COVID-19 disease were either overweight (33.3%) or obese (66.67%). Multiple regression analysis showed that high BMI, especially obesity, was a prognostic factor for severe disease in COVID-19 infection in young people (44).

Bartoletti et al. studied 1,265 inpatients diagnosed with COVID-19 in 11 Italian hospitals from February to April 2020. Multiple logistic regression analysis indicated that obesity was associated with severe respiratory failure, after adjustment for other variables (aQR 4.62; 95% CI 2.78–7.70) (60).

Mughal et al. in a retrospective study involving the first 129 patients with COVID-19 admitted to the Monmouth Medical Center (U.S.) from March to April 2020, concluded that among the patients who received IMV, a higher proportion was obese in comparison with those who did not require such intervention (36.7 vs. 10.1%, *p* = 0.0334) (61).

Sahin et al. reported that, in a total of 14,625 patients of median age 42years, presenting with COVID-19 between March and May 2020, mortality was significantly higher in obese or overweight patients. On adjusted analysis, overweight (OR, 95% CI: 1.82, 1.04–3.21; *p* = 0.037) and obesity (OR, 95% CI: 2.69, 1.02–1.05; *p* < 0.001) were associated with a higher rate of intubation/mechanical ventilation (34).

Similarly, Hendren et al. in a study of 7,606 patients with COVID-19, showed that overweight patients and obese (classes I to III) patients were at higher risk for mechanical ventilation [OR 1.28 (95% CI, 1.09–1.51), 1.54 (1.29–1.84), 1.88 (1.52–2.32), and 2.08 (1.68–2.58), respectively] (52).

Bailly et al. showed that IMV was necessary more frequently for obese in-patients with COVID-19 (aOR 1.9, 95% CI 1.8–2.0) (62). Yoshida et al. in a retrospective study of 776 patients, reported that obesity was a predictor of respiratory failure requiring IMV at a lower BMI class (> 35 kg/m^2^) in women (41). Conversely, Le Guen et al. in a retrospective cohort study that enrolled 600 obese patients who were positive for COVID-19, reported that the intubation rate (*p* = 0.3705) was not significantly higher in obesity (37).

### Obesity in Patients With COVID-19 and Mortality

Palaiodimos et al. reported that 24% of 200 patients with COVID-19 in their retrospective study died during hospitalization, and that the mortality rate was higher in patients with severe obesity, specifically, BMI < 25 kg/m^2^: 31.6%, BMI 25–34 kg/m^2^: 17.2%, BMI ≥ 35 kg /m^2^: 34.8%, *p* = 0.03 (56). Similar results were reported by Van Halem et al. who studied 319 patients aged 16 years or older, hospitalized for at least 24 hours with confirmed COVID-19 infection by April 15 at a tertiary care center. They reported that 33.9% of patients who died were obese and that the percentage of obese people discharged was 20.11% (*p* = 0.039) (63).

Conversely, Busetto et al. reported that the mortality rate was significantly higher in the normal weight group (31.2%) than in overweight patients (no death) or in obese patients (6.9%) (p <0.001) (32).

In the study of Nakeshbandi et al. overweight (aOR 1.4, 95% CI 1.1–1.9, *p* = 0.003) and obese patients (aOR 1.3, 95% CI 1.0–1.7, *p* = 0.04) had an increased risk of mortality compared with those of normal BMI (58). Similarly, Klang et al. demonstrated on multiple regression analysis that in both the younger population (<50 years, *n* = 572) and the elderly population (> 50 years, *n* = 2,834), BMI ≥ 40 kg/m^2^ was independently associated with mortality (aOR 5.1, 95 CI: 2.3–11.1 and aOR 1,6; 95% CI: 1.2–2.3, respectively) (59).

Czernichow et al. in a study of 5,795 patients with COVID-19 hospitalized in a Network Hospital Assistance Publique—Hôpitaux in Paris, concluded that mortality was significantly higher in obese individuals, specifically, BMI 30–35 kg/m^2^, aOR 1.89; 95% CI: 1.45–2.47; BMI 35–40 kg/m^2^, aOR 2.79; 95% DE: 1.95–3.97 and BMI > 40 kg/m^2^, aOR 2.5; 95% CI: 1.62–3.95, compared with a control group of BMI 18.5–25 kg/m^2^ (64).

In Mexico, also, a study conducted by Prado-Galbarro et al. which included all consecutive cases of COVID-19 treated by medical units and hospitals in Mexico between February and April 2020 (*n* = 15,529), demonstrated that obesity was associated with a higher risk of mortality from infection SARS-CoV-2 (in out-patients aOR: 1.55; 95% CI: 1.42–1.51), and in in-patients aOR: 12.84; 95% CI: 1.15–7.00) (65).

In the retrospective study of Chetboun et al. 1,461 patients were enrolled, with a median BMI of 28.1 kg/m^2^ (IQ range 25.4–32.3 kg/m^2^). An adjusted Cox proportional hazards regression model demonstrated a significant association between BMI and death, which was only increased in the class III obesity category [BMI ≥ 40 kg/m^2^; hazard ratio = 1.68 (95% CI: 1.06–2.64)] (66).

The research findings of Sahin et al. were similar; they enrolled 14,625 patients with COVID-19 of median age 42 years of whom 57.4% were female, categorized into three groups, normal weight (34.7%), overweight (35.6%), and obese (29.7%). Obesity in this population was associated with increased mortality (OR, 95% CI: 2.56, 1.40–4.67; *p* = 0.002) (34).

Hendren et al. analyzed data from 7,606 patients hospitalized with COVID-19 at 88 US hospitals and concluded that classes I and II obesity were associated with a higher risk of in-hospital death [OR 1.28 (95% CI, 1.09–1.51), and 1.57 (1.29–1.91), respectively], and class III obesity was also associated with a higher risk of in-hospital death [hazard ratio, 1.26 (95% CI, 1.00–1.58)] (52).

Tartof et al. among 6,916 patients with COVID-19, compared patients with a BMI of 18.5 to 24 kg/m^2^ with those with a BMI of 40–44 and ≥ 45 kg/m^2^. In adjusted analysis, high BMI was strongly associated with a higher mortality risk, with a 4 times greater risk for the highest BMI classes. The adjusted mortality rate for the highest BMI classes was 7.08 per 100 patients (95% CI 3.58–14.00), equal to an attributable excess of 5.52 deaths per 100 patients (95% CI 0.63–10.42) when compared with those of BMI 18.5–24 kg/m^2^ (67).

Azarkar et al. analyzed 364 cases of COVI-19, from February to September 2020, and reported that mortality showed a significant relationship with BMI (*p* < 0.05) (68).

In a retrospective cohort study conducted by Frank et al. a total of 305 patients were categorized by BMI: <25 kg/m^2^, 54 patients (18%), ≥ 25– < 30 kg/m^2^, 124 patients (41%), ≥ 30 kg/m^2^– <35 kg/m^2^, 58 patients (19%), and ≥35 kg/m^2^, 69 patients (23%). In total, 128 patients (42%) had a severe disease course; 119 (39%) with intubation, and 9 (3%) died without intubation. Furthermore, 65 patients (51%) with BMI ≥ 30 kg/m^2^ were intubated or died. Adjusted Cox models showed that BMI ≥ 30 kg/m^2^ was associated with a 2.3-fold increased risk of intubation or death (95% CI, 1.2–4.3) compared with individuals with BMI <25 kg/m^2^ (69).

Richardson et al. in a retrospective cohort study analyzed the records of 1,013 patients in March and April 2020. MR analysis revealed that obesity was an independent predictor of in-hospital 30-day mortality [adjusted hazard ratio (aHR) 2.71, 95% CI 1.28–5.73; *p* = 0.002] (70).

Smati et al. in the retrospective, multicenter, nationwide CORONADO study, assessed the relationship between BMI classes and early COVID-19 prognosis in 1,965 patients with type II diabetes mellitus (DM). MR analysis showed significant association between poor prognosis and overweight [OR 1.65 (1.05–2.59)], class I [OR 1.93 (1.19–3.14)], and class II/III obesity [OR 1.98 (1.11–3.52)] (*p* = 0.0373). An association was also found between IMV and overweight to class II/III obesity, but death was not associated with BMI status (*p* = 0.9634) (71).

Finally, a retrospective study of the COVID-19 ICU Group by Schmidt et al. in Switzerland involving all consecutive patients (*n* = 4,643) aged ≥16 years of age admitted to the ICU between February and May 2020, with laboratory-confirmed ARDS due to SARS-CoV-2 (*n* = 4,244) concluded that severe obesity, BMI ≥ 40 kg/m^2^, is one of the foremost prognostic indicators of death during the 90 days, regardless of other variables in the model (aOR: 2.05; 95% CI 1.28–3.27) (72).

The relationship between obesity and mortality was not confirmed in the study of Le Guen et al. in which obese patients showed no differences in mortality (*p* = 0.248) (37).

Suresh et al. enrolled 1,983 patients of whom 1,031 (51.9%) were obese and 952 (48.9%) were not obese. The obese patients were younger than the patients of normal weight (*p* < 0.001). MR models adjusting for differences in sex, race, age, medical comorbidities and treatment modalities revealed no difference in 60-day mortality and 30-day readmission between the groups with and without obesity. The obese patients showed increased odds of ICU admission (aOR 1.37; 95% CI, 1.07–1.76; *p* = 0.012) and intubation (adjusted OR 1.37; 95% CI, 1.04–1.80; *p* = 0.026) (73).

## Discussion

Numerous studies in diverse populations from around the world have examined the role of body weight and body composition in relation to COVID-19 disease. Studies investigating the effects of BMI were thoroughly researched in the context of our review, yielding important conclusions. It appears that a high BMI is an indicator for the development of severe COVID-19 disease, as assessed by admission to the ICU, intubation, and even mortality. The results suggest the significance of maintaining a healthy body weight as a preventive measure against severe infection.

Basic research has shown that adipose tissue, and especially visceral fat, functions as an active gland, and is implicated in subclinical inflammation, *via* to the release of lipocytokines. The profile of lipocytokines changes in obesity, favoring inflammation, with activation of pro-inflammatory monocytes. The result is a vicious cycle of positive feedback, where inflammation and metabolic dysfunction reinforce each other (74). This mechanism is particularly important in COVID-19 disease, in which an exaggerated inflammatory response leads to severe disease (75).

This review had some limitations, mainly concerning the effect of the different virus strains that were responsible for the infection in the patients. Since the evolution of the pandemic different variants of SARS-CoV-2 revealed that were associated with one or more changes at the degree of global health significance, mainly affecting: transmissibility, changes in COVID epidemiology, increase in virulence or change in disease presentation, decrease in effectiveness of public health and social measures or available diagnostics, vaccines and therapeutics. Unfortunately, during in the studies included in our review the specific information on the variants was not reported. Nevertheless, throughout the 2-year period that we revised, obesity was linked with increased complications, hospitalization and mortality rates, regardless the predominant variant reported in each sub-period. In addition, in the various studies reviewed, diverse populations were recruited and different factors were examined by different researchers, while in many cases the classification of BMI varied or subjects were classified only on the presence or absence of obesity. Finally, micronutrients' deficiencies, that contribute to the severity of COVID-19 symptoms in obese or inactive subjects were not investigated herein. For instance, vitamin D is a stakeholder for erasing inflammatory complications of COVID-19 (76, 77). Specifically in obese subjects with vitamin D deficiency (78), that more often occur during winter (79, 80) that COVIV-19 infection arose, increases the risk factors for COVID-19 mortality (81). For all the above reasons, we cannot draw definitive conclusions on the effect of the factors examined.

## Conclusion

To conclude, despite the limitations of this review, it highlights the importance of body weight for the complications and prognosis of COVID-19 infection, and it should be taken into consideration in clinical practice. Obesity in patients with COVID-19 is independently associated with an increased risk of ICU admission, intubation and death. Recognizing that obesity impacts morbidity and mortality in this manner is crucial for appropriate management of patients with COVID-19 and probably expand the accumulated knowledge in the management of other infectious diseases.

In the future, animal studies or *in vitro* experiments on cells need to be performed to reveal the possible molecular mechanisms involved. Investigation is also needed on the effect of BMI on vaccination response/protection.

## Author Contributions

EV: conception and design of the work, data collection and interpretation, and drafting the article. AKP and ANP: data collection and interpretation. RB and DB: data collection and interpretation and critical revision of the article. All authors approved the final version.

## Conflict of Interest

The authors declare that the research was conducted in the absence of any commercial or financial relationships that could be construed as a potential conflict of interest.

## Publisher's Note

All claims expressed in this article are solely those of the authors and do not necessarily represent those of their affiliated organizations, or those of the publisher, the editors and the reviewers. Any product that may be evaluated in this article, or claim that may be made by its manufacturer, is not guaranteed or endorsed by the publisher.
