# Supplementary material for: Obesity and Infection: What Have We Learned From the COVID-19 Pandemic
Source: Front Nutr. 2022 Jul 22;9:931313. doi: 10.3389/fnut.2022.931313 (PMC9353573; doi:10.3389/fnut.2022.931313)
Supplement: Supplementary file 1 [file Table_1.pdf]

Table 1 Characteristics of Studies in the review of obesity and severity of COVID-19 infection and mortality (n=44)

**Authors, date, (reference) Country**	**Type of study**	**Population**	**Independent variable**	**Outcomes**
Alkhatib et al, 2020<br>(28)<br><br>USA	Retrospective, cross-sectional	158 African American patients with COVID-19 treated between 12 March and 9 April 2020, at a single tertiary center.<br><br>Men 38.6%<br>Mean age 57 years	BMI classes<br><br>Normal weight <30 (n=62)<br>Overweight 30-35 (n=37)<br>Obese 35-40 (n=37)<br>Severe obesity >40 (n=22)	Patients admitted to ICU were older (62 vs. 55 years, P = 0.003) and had higher BMI (36.5 vs 31.9, p = 0.002). BMI was a significant determinant of ICU admission in African Americans:<br>Increase in BMI by 5 kg/m² and 10 kg/m²increased probability of ICU admission: OR 1.72 and 2.97, respectively*
Rao et al, 2020<br>(43)<br><br>China (Wuhan)	Retrospective cohort study	240 patients with COVID-19 admitted to Union Hospital in Wuhan, China, between 24 December 2019and 25 March 2020.<br><br>Men 46.2%<br>Median age 48 years	BMI classes<br><br>Normal weight 18.5–23.9<br>Overweight 24.0–27.9	Patients with severe disease had significantly higher BMI level and were more likely to be overweight [73 (60.8%) vs 41 (34.2%), p<0.001]. Overweight patients more likely to develop severe pneumonia than normal-weight patients. [73 (64.0%) vs 47 (37.3%), p<0.001]. Overweight was an independent risk factor for severe pneumonia (OR 3.075, p = 0.021)*
Palaiodimos et al, 2020<br>(29)	Retrospective study	200 patients with COVID-19 admitted to a tertiary medical center.<br><br>Men 51%	BMI classes<br><br>Normal weight <25<br>Overweight 25-34<br>Obese >34	24% of patients died, with higher rates in those with severe obesity (BMI < 25: 31.6%, BMI 25–34: 17.2%, BMI ≥ 35: 34.8%, p = 0.030).

USA (New York)		Median age 64 years		Patients with severe obesity more likely to undergo intubation (BMI < 25: 18.4%, BMI 25–34: 16.4%, BMI ≥ 35: 34.8%, p = 0.032).<br>45% had increasing oxygen requirements, with no significant difference between BMI groups22% developed ARDS and 16% spent at least one night in the ICU.<br>BMI ≥ 35 was associated with in-hospital mortality (OR 3.78, p = 0.006), increasing oxygen requirements (OR 3.09, (p = 0.004) and intubation (OR 3.87,p = 0.006)*.
Simmonet et al, 2020 (57)<br><br>France	Retrospective cohort study	124 consecutive patients admitted to ICU with SARS-CoV-2 between 27 February 2020 and 5 April 2020<br><br>Men 73%<br>Median age 60 years	BMI classes<br><br>Normal weight <25 (n=17)<br>Overweight 25-30 (n=48)<br>Obese 30-35 (n=24)<br>Severe obesity >35 (n=35)	Obesity and severe obesity were present in 47.6% and 28.2% of cases, respectively. 85 patients (68.6%) required IMV. The proportion of patients who required IMV increased with BMI category (p < 0.01). OR for IMV in patients with BMI >35 vs patients with BMI <25 was 7.36 (p = 0.02)*
Nakeshbandi et al, (2020 (58)<br><br>USA (New York)	Retrospective cohort study	504 patients who tested positive for COVID-19 in a COVID-only hospital, between 10 March and 13 April 2020.<br><br>Men: 52%<br>Mean age: 67 years	BMI classes<br><br>Normal weight 18.50–24.99, n = 139 (27%)<br>Overweight 25.00–29.99, n = 150 (30%)<br>Obese ≥ 30.00, n = 215 (43%)	Significant increase in risk of mortality in overweight (RR 1.4) and obese (RR 1.3), compared with those with normal BMI*. Significant increase in risk of intubation in overweight (RR 2.0) and obese (RR 2.4) compared with those with normal BMI*. Obesity did not affect the rates of AKI, ACI, or ARDS.

Cai et al, 2020 (45)<br><br>China	Retrospective multicenter clinical study	96 patients hospitalized with SARS-CoV-2 infection between 23 January and 14 February 2020.<br><br>Men: 52.5%<br>Mean age: 35.4 years	BMI classes<br><br>Normal weight <24<br>Overweight 24-27.9<br>Obese ≥28	Proportion of patients with ARDS with BMI < 24, 24–27.9, and ≥ 28 were 52%, 24%, and 24%, respectively, and significantly different from 64.8%, 29.6% and 5.6% in patients without ARDS (p= 0.035). After treatment, 66 patients showed improved or stable disease (mean BMI 22.35 ± 3.56), 30 showed worsening (mean BMI 24.89 ± 3.17) (p= 0.001). Proportion with negative chest CT was 85.7%, 14.3% and 0% for patients with BMI < 24, 24– 27.9 and ≥ 28, respectively, and of patients with pneumonia, 54.7%, 32% and 13.3%, respectively (p= 0.027). The mean BMI 20.78 ± 3.15 in patients without, vs 23.81 ± 3.49 in patients with pneumonia (p= 0.001).
Denova-Gutierrez et al, 2020 (46)<br><br>Mexico	Cross-sectional study	3,844 Patients positive for COVID-19 according to the Mexican National Epidemiological Surveillance System from 27 February 2020 to 10 April 2020.<br><br>Men:<br>COVID-19 positive: 42%,<br>COVID-19 negative: 58%<br>Mean age:<br>COVID-19 positive: 45.4 years<br>COVID-19 negative: 38.8 years	Obesity condition<br><br>Without obesity (n=3,178)<br>With obesity (n=668)	Patients with COVID-19 were classified according to obesity: 17.4% were obese. A higher proportion of patients with obesity required ICU support and IMV. After adjusting for other factors, obese patients showed 1.43-fold higher odds of developing severe COVID-19 than non-obese patients*.

Busetto et al, 2020<br>(32)<br><br>Italy	Retrospective cohort study	92 patients hospitalized in the medical COVID-19 ward with severe ARDS from 23 March to 11 April 2020<br><br>Men: 61.9%<br>Mean age: 57 years	BMI classes<br><br>Normal weight <25<br>Overweight ≥25, <30<br>Obese ≥ 30.00	Assisted ventilation (NIV or IMV) beyond pure oxygen support was used in 15.6% of normal weight patients, 54.8% of overweight patients and 41.4% of obese patients ($p < 0.01$). ICU or semi-intensive respiratory unit was required for 18.7% of normal weight patients, 54.8% of overweight patients and by 41.3% of obese patients ($p < 0.05$). The mortality rate was significantly higher in the normal weight patients (31.2%) than in overweight (no deaths) or obese patients (6.9%) ($p < 0.001$).
Dreher et al, 2020<br>(35)<br><br>Germany	Retrospective cohort study	The first 50 patients hospitalized at the Aachen University Hospital in February and March 2020 who tested positive for SARS-CoV-2<br><br>Men: 34%<br>Median age: 50 years	BMI classes<br><br>Overweight ≥25, <30<br>Obese ≥30	Patients who developed ARDS were more frequently obese (46% vs 23%) or overweight (38% vs 19%) than those without ARDS.
Wang et al, 2020<br>(42)<br><br>China (Jiangsu)	Retrospective study	297 patients diagnosed with COVID-19, between January 18 and February 26 2020, enrolled from 10 medical centers in 10 cities<br><br>Median age:<br>lean 38 years<br>overweight 48 years<br>obese 47 years	BMI classes<br><br>Lean <24 (n=140)<br>Overweight ≥24 BMI, < 28 (n=117)<br>Obese ≥28 (n=40)	BMI distribution differed between patients with severe and non -severe illness ($p < 0.001$). Severe illness more frequent in overweight and obese than in lean patients (12.82% vs. 2.86%, $p = 0.006$, and 25% vs. 2.86%, $p < 0.001$, respectively). ICU admission comparable among the three groups.

				Duration of hospitalization longer in obese than lean patients (17 days vs 14 days, p = 0.029).<br>Overweight (OR 4.222) and obesity (OR 9.216) were independent risk factors for severe COVID-19 after adjustment*.
Kalligeros et al (2020)<br>(30)<br><br>Greece	Retrospective cohort study	103 patients hospitalized with COVID-19 in Rhode Island between 17 February and 5 April 2020<br><br>Men: 61.2%<br>Median age: 60 years	BMI classes<br><br><25: 19 (18.4%)<br>25 -2 9.9: 35 (33.9%)<br>30 - 34.9: 22 (21.3%)<br>>34.9: 27 (26.2%)	During the first 10 days of hospitalization, 44/103 were admitted to the ICU, and 29 required IMV. The prevalence of obesity was 47.5% in hospitalized patients, 56.8% in those admitted to ICU and 65.5% in those requiring IMV. Only severe obesity (≥35) was significantly associated with ICU admission (aOR: 5.39)*.
Klang et al, 2020<br>(59)<br><br>USA (New York City)	Retrospective study	3,406 patients with COVID-19 hospitalized in a large academic hospital system between 1 March and 17 May 2020.<br><br>Men: 69.4%<br>Median age: 43.25years	BMI classes<br><br>Normal weight <30<br>Obese 30-40,<br>Severely obese ≥40	BMI ≥40 was independently associated with mortality, in patients aged <50 years (n=572): aOR 5.1, and in those aged ≥ 50 years:(aOR 1.6)*<br>Intubation and IMV independently associated with BMI ≥40, in both younger (aOR 4.1) and older age group (aOR 1.5)*
Czernichow et al, 2020<br>(64)<br><br>France (Paris)	Retrospective study	All patients (n = 5,795) with ARDS due to COVID-19, hospitalized between 1 February and 30 April 2020.<br><br>Men: n=2,004<br>Mean age: 59.6 years	BMI classes<br><br>Underweight < 18.5<br>normal weight 18.5-25<br>25-30<br>30-35<br>35-40<br>>40	ICU admission rates increased proportionally to BMI categories. IMV did not follow an obvious trend according to BMI category. Mortality significantly higher in obese individuals: OR 1.89, 2.79 and 2.55 for BMI 30-35, 35-40 and

				>40, respectively, compared to BMI 18.5-25 as a reference category*
Hu et al, 2020<br>(47)<br><br>China<br>(Wuhan)	Retrospective study	323 patients hospitalized with COVID-19 from 8 January to 20 February 2020<br>Men 51.4%<br>Median age 61 years	BMI classes<br><br><25<br>25-30<br>>30	Obese patients (BMI ≥ 30) were more likely to have an unfavorable than a positive clinical outcome (10.7% vs 2.9%, p = 0.029)
Van Halem et al, 2020<br>(63)<br><br>Belgium	Retrospective study	319 patients aged ≥16 years admitted to hospital for at least 24 hours with confirmed COVID-19, up until 15 April 2020<br><br>Men 68.93%<br>Median age 74 years	BMI classes<br><br>Underweight <18.5<br>Normal weight 18.5-25<br>Overweight 25-30<br>Obese >30	40% of patients were overweight and 23% were obese. Overall case fatality 25% of whom 33.9% were obese (p = 0.039).
Cai et al, 2020<br>(48)<br><br>China<br>(Shenzhen)	Retrospective study	383 patients hospitalized with COVID-19 admitted from 11 January to 16 February 2020 and followed until 26 26 March 2020<br><br>Men 47.78%<br>Median age 53years	BMI classes<br><br>Underweight <18.5<br>Normal weight 18.5-23.9<br>Overweigh: 24-27.9<br>Obese >28	91/383 patients (23.8%) progressed to severe COVID-19. No underweight patient developed severe COVID-19. 39 (19.2%) severe cases were normal weight, 36 (29.3%) overweight and 16 (39%) obese.<br><br>3 patients (0.78%) died of COVID-19, one (0.49%) of normal weight, one (0.81%) overweight group, and one (2.44%) obese.<br>In comparison with those of normal weight, patients who were overweight or obese had a higher risk of developing severe disease

				(OR 1.84 and OR 3.4, respectively)*
Deng et al, 2020<br>(44)<br><br>China (Wuhan)	Retrospective cohort study	65 consecutively admitted patients with COVID-19, aged between 18 and 40 years between 13 March and 12 April 2020,	BMI classes<br><br>Normal weight <24<br>Overweight 24-28<br>Obese >28	Severely ill patients were either overweight or obese Subcutaneous fat thickness had no detectable effect on the severity of disease.
Smati et al,2020<br>(71)<br><br>USA	Retrospective study	1,965 patients from the CORONAvirus-SARS-CoV-2 and Diabetes Outcomes (CORONADO) study T2DM.<br><br>Men 64.5%<br>Mean age 70.1 years	BMI classes<br><br>Normal 18.5-24.9<br>Overweight 25-29.9<br>Class I obese 30-34.9<br>Class II obese ≥35	By the 7th day (D7), 385 (19.6%) patients required IMV, 190 (9.7%) died and 388 (19.8%) patients were discharged.<br>IMV and/or death by D7 was significantly associated with overweight, class I and class II/III obesity (OR 1.65, 1.93, 1.98, respectively, p = 0.0373)*
Ioannou et al, 2020<br>(49)<br><br>USA	Retrospective study	10,131 patients positive for SARS-CoV- 2 in the Veterans Affairs (VA) national health care system (11.4%) between 1 March and 14 May , 2020<br><br>Men 91%<br>Mean age 63.6 years	BMI classes<br><br>Underweight <18.5<br>Normal weight 18.5-24.9<br>Overweight 25-29.9<br>Obese I 30-34.9<br>Obese II ≥35	Compared with underweight and normal weight, patients with Class I and Class II obesity had a higher risk of IMV and mortality

Nachega et al, 2020<br>(50)<br><br>DR Congo (Kinshasa)	Retrospective cohort study	766 confirmed COVID-19 cases, between 10 March and 31 July 2020 in 7 hospitals<br><br>Men 91%<br>Median age 63.6 years	Obesity<br><br>With obesity (n=39)<br>Without obesity (n=72)	Obese patients were less likely to improve than non-obese patients (aOR 0.27)*
Fresan et al, 2020<br>(51)<br><br>Spain	Prospective population-based cohort study	433,995 persons with COVID-19 in Spain during March and April of 2020.<br><br>Age 25-79 years	Obesity<br><br>Without morbid obesity (n=426,535)<br>With morbid obesity (class 3) (n=7,460)	1,105 were hospitalized for confirmed COVID-19 (255 per 100,000 inhabitants)<br>176 had severe disease (41 per 100,000) 117 were admitted to the ICU and 97 died (28 in the ICU). Subjects with class 3 obesity made up 1.7% of the study population, 4.7% of the COVID-19 hospitalizations and 5.7% of the severe cases. Severe COVID-19 (ICU admission/death) was twice as frequent in persons with class 3 obesity as in the rest of the population (aOR: 2.30, p = 0.012)*
Bartoletti et al, 2020<br>(60)<br><br>Italy	Multicenter cohort study	1,265 patients diagnosed with COVID-19 from 22 February to 3 April 2020, hospitalized >24 hours at 11 Italian hospitals.<br><br>Men 63.3%<br>Mean age 65.7 years	Obesity<br><br>Yes/No	Obesity was a risk factor for severe respiratory failure (OR 4.62)*

Shuelter-Trevisol et al, 2020 (31)<br><br>Brazil	Prospective cohort study	211 hospitalized patients diagnosed with COVID-19 between 16 March 16 and 16 July 16 2020, in the two hospitals with ICUs in Tubarão, Santa Catarina, Brazil.<br><br>Men: 53.6%	Obesity<br><br>Yes/No	Obesity, chronic pulmonary diseases, and advanced age were independent risk factors for worsening clinical condition requiring ICU admission (OR 6.83)*
Yanover et al, 2020 (55)<br><br>Israel	Retrospective study	4,353 people testing positive for SARS CoV-2<br><br>Men 45.5%<br>Median age 35 years	Obesity<br><br>Yes/No	Obese patients more likely to have severe outcome (42.2% vs 19.2%)
Prado-Galbarro et al, 2020 (65)<br><br>Mexico	Observational study	15,529 patients with COVID-19 in Mexican healthcare units and hospitals between 27 February and 27 April 2020.<br><br>Men: 57.8%<br>62.6% aged ≥ 40 years	Obesity<br><br>Yes/No	Obesity was associated with a higher risk of mortality after SARS-CoV-2 infection<br>in outpatients (OR 1.55 and<br>in hospitalized patients (OR 12.84)*

Mughal et al, 2020<br><br>(61)<br>USA	Monocentric retrospective study	The first 129 patients with COVID-19 admitted to Monmouth Medical Center from 1 March 1 to25 April 2020<br><br>Men 47.2%<br>Mean age 63.0 years	Obesity<br><br>Yes/No	Percentage of patients with obesity higher in patients who received IMV than those who did not (36.7% vs 10.1%, p = 0.0334).<br>The incidence of obesity was no different in those who successfully exited IMV and those who did not
Argenzian et al, 2020<br>(33)<br><br>USA (New York)	Retrospective manual medical record review	The first 1,000 patients presenting at the ED admitted to hospital between<br>1 March and 5 April 2020<br><br>Men 596<br>Median age 63.0 years	Obesity<br><br>Yes/No	Hospitalized patients, particularly those treated in ICU, were more often obese (45.7%) and had baseline comorbidities including hypertension and DM (39.5%)
Schmidt et al (2021)<br><br>(72)<br>France, Switzerland, Belgium	Prospective cohort study	All consecutive patients aged >16 years of age admitted to ICU with severe SARS-CoV-2) infection<br><br>Men 54%<br>Median age 63.0 years	Obesity<br><br>Yes/No	Severe obesity ≥40 was a predictor of 90-day mortality (OR 2.05)*

Sahin et al, 2022<br>(34)<br><br>Turkey	Retrospective study	14,625 patients enrolled between 11 March and 30 May 2020<br><br>Median age 42 years	BMI classes<br><br>Normal weight 18.5-23.9<br>Overweight 24-27.9<br>Obese >28kg	Hospitalization, ICU admission, intubation/mechanical ventilation, pulmonary involvement and mortality were significantly higher in patients who were overweight or obese. Overweight and obesity were associated with intubation/IMV (OR1.82, 2.69, respectively,1.02–1.05; p < 0.001)* Only obesity was associated with increased mortality (OR 2.56, p = 0.002)*.
Le Guen et al, 2021<br>(37)<br><br><br><br>USA (North Philadelphia)	Retrospective cohort study	600 patients who were positive for COVID-19 between 15 March 6 May 6 2020.	BMI classes<br><br>Underweight <18.5<br>Normal weight 18.5-25<br>Overweight 25-30<br>Obese I 30-35<br>Obese II 35-40<br>Obese III ≥40	Patients with obesity had an increased rate of ICU admission (p=0.0215) and increased length of stay (p=0.0004), but no difference in intubation rate (p=0.3705) or mortality (p=0.2486).

Gao et al, 2021<br>(36)<br><br>England	Prospective cohort study	6,910,695 with a positive test for SARS-CoV-2 between 24 January and 30 April 2020<br><br>Mean BMI 26.78	BMI classes<br><br>Normal weight 18.5-24.9<br>Overweight 25-29.9<br>Obese I 30-34.9<br>Obese II ≥35	J-shaped association between BMI and admission to hospital due to COVID-19 and death (aHR f 1·05 and 1·04, respectively)*, and a linear association with ICU admission across the whole BMI range
Hendren et al, 2021<br>(52)<br><br>USA	Cohort study	7,606 patients hospitalized with COVID-19 at 88 US hospitals enrolled in the AHA COVID-19 Cardiovascular Disease Registry, with data collection through 22 July 2020	BMI classes<br><br>Underweight <18.5<br>Normal weight 18.5-25<br>Overweight 25-30<br>Obese I 30-35<br>Obese II 35-40<br>Obese III >40	Obesity classes I to III associated with in-hospital death and IMV (OR 1.28 and 1.80, respectively)*. Class III obesity associated with a higher risk of in-hospital death (HR 1.26)*. Higher likelihood of MVI in overweight and class I to III obesity (OR 1.28, 1.54 and 2.08, respectively)*. Class III obesity associated with increased risk of in-hospital death only in those aged ≤50 years (HR 1.36)*

Cordova et al, 2021<br>(40)<br><br>Argentina	Multicenter prospective cohort study	809 patients admitted to a center participating in the ECCOVID study, with SARS-CoV-2 infection confirmed and enrolled between 3 March and 15 October 15 2020<br><br>Men 56%<br>Median age 53 years,	Obesity<br><br>Yes/No	Obesity was independently associated with ICU admission (OR 2.38)*, hypertension (OR 3.21)* and oxygen saturation ≤93% (OR 6.45)*
Pantea Stoian et al, 2020<br>(53)<br><br>Roumania	Retrospective study	432 patients in the Romanian COVID-19 mortality substudy<br><br>Mean age 67 years	Obesity<br><br>Yes/No	Obesity is a negative marker for severity of COVID-19 infection in adults aged <50 years (p = 0.0001)

Tartof et al, 2020<br>(67)<br><br>USA (Southern California)	Retrospective cohort study	6,916 patients identified with COVID-19 in Kaiser Permanente system between 13 February and 2 May 2020	BMI classes<br><br>Underweight <18<br>Normal weight 18.5-24<br>Overweight 25-30<br>Obese I 30-35<br>Obese II 35-40<br>Obese III 40-44<br>Extreme obesity ≥45	High BMI strongly associated with higher risk in mortality, with at least 4 times the risk for the highest BMI measures. Adjusted mortality rate 7.08/100 patients, equal to an attributable excess of 5.52/100 patients compared with BMI 18.5-24
Terada et al, 2021<br>(54)<br><br>Japan	Cohort study	3,376 cases of COVID-19 from 16 January to 31 May 2020<br><br>Median age<br>non severe (n=2,196) 50 years<br>severe (n=1,180) 67 years	Obesity<br><br>Yes/No	Obesity had major effect on worst severity (OR 1.75, p = 0.001)*

Chetboun et al (2021)<br>(66)<br><br>International Europe, Israel, USA	Retrospective international multicenter study	1,461 patients admitted to ICU for COVID-19 in 21 centers between 19 February and 19 May 2020.<br><br>Men 73.2%<br>Median age 64 years	BMI classes<br><br>Underweight <18.5<br>Normal weight 18.5-25<br>Overweight 25-30<br>Obese I 30-35<br>Obese II 35-40<br>Obese III 40-44	Linear relationship between BMI per 5 kg/m² and IMV (OR 1.27)* (95% CI: 1.12- 1.45). Association between BMI and mortality only in obesity class III*
Suresh et al, 2021<br>(73)<br><br>USA (Michigan)	Retrospective cohort study	1,983 adult patients with COVID-19 hospitalized between 1 March 1 and 30 April 2020	Obesity<br><br>Yes/No	Obese patients were younger than patients with normal weight (p < 0.001). No difference in 60 day mortality and 30 day readmission between groups with obesity and without obesity, but obese patients had increased odds of ICU admission and intubation (aOR 1.37, p= 0.012, and 1.37, p = 0.026, respectively)*

Boudou et al, 2021 (39)<br><br>Ireland	Retrospective study	47,265 laboratory-confirmed cases of symptomatic COVID-19 infection from 29 February to 30 November 2020<br><br>Mean age 41.2 years	Severe obesity (BMI ≥ 40)<br><br>Yes/No	Severe obesity was a significant marker for ICU admission (OR 19.6)*, and a significant predictor in patients aged < 41 years and < 63 years for ICU admission and death, respectively
Bailly et al 2022 (62)<br><br>France	Retrospective cohort study	134,209 patients, admitted to hospital for COVID-19between February and September 2020.	Obesity<br><br>Categorized in the preceding decade preceding admission for COVID-19 as<br>No obesity<br>Obesity<br>Prevalent obesity<br>Incident obesity	IMV was more frequent for obese inpatients (aOR 1.9)*

Yoshida et al, 2021<br>(41)<br><br>USA (New Orleans, LA)	Retrospective study	776 adult patients hospitalized for COVID-19 at two tertiary care academic hospitals from 27 February to 15 July 2020<br><br>Women 61.4%<br>Median age 60.5 years	BMI classes<br><br>Underweight <18.5<br>Normal weight 18.5-25<br>Overweight 25-30<br>Obese I 30-35<br>Obese II 35-40<br>Obese III ≥40	Obesity was independently associated with increased odds of IMV and ICU admission.<br>Obesity was a predictor of respiratory failure requiring IMV at a lower BMI (> 35) in women.
Azarkar et al, 2021<br>(68)<br><br>Iran	Retrospective cohort study	364 cases of COVID-19 from February to September 2020<br><br>Mean age 54.28±18.81 years	BMI classes<br><br>Normal weight < 25 (54 patients, 18%)<br>Overweight 25 - ≤30 (124 patients, 41%)<br>Obese I 30 ≤35 (58 patients, 19%)<br>Obese II ≥ 35 (69 patients, 23%)	Mortality was significantly associated with BMI ($p<0.05$).<br>119 patients (39%) needed intubation and 9 (3%) died.<br>65 patients (51%) with BMI ≥30 kg/m2 were intubated or died.

Frank et al, 2020<br>(69)<br><br>USA (Massachusetts)	Retrospective cohort study	305 consecutive patients hospitalized with confirmed COVID-19between 13 March and 3 April 2020<br><br>Mean age 60 years	Obesity<br><br>Yes/No	BMI ≥30 associated with a 2.3-fold increased risk of intubation or death compared with BMI <25*
Richardson et al, 2021<br>(70)<br><br>USA (New York City)	Retrospective cohort study	1,013 patients with COVID-19 hospitalized in 12 acute care hospitals between 1 March and 27 April 2020 in the New York City area.	BMI classes<br><br>Normal weight 18.5-25<br>Overweight 25-29.9<br>Obesity I 30-39.9<br>Severe obesity ≥40	Obesity was independent predictor of in-hospital 30-day mortality (aHR 2.71, p = 0.002).
